# NIST High Accuracy Reference Reflectometer-Spectrophotometer

**DOI:** 10.6028/jres.101.061

**Published:** 1996

**Authors:** James E. Proctor, P. Yvonne Barnes

**Affiliations:** National Institute of Standards and Technology, Gaithersburg, MD 20899-0001

**Keywords:** bidirectional, diffuse, hemispherical, monochromator, reflectance, reflectometer, scatter, spectrophotometer, specular

## Abstract

A new reflectometer-spectrophotometer has been designed and constructed using state-of-the-art technology to enhance optical properties of materials measurements over the ultraviolet, visible, and near-infrared (UV-Vis-NIR) wavelength range (200 nm to 2500 nm). The instrument, Spectral Tri-function Automated Reference Reflectometer (STARR), is capable of measuring specular and diffuse reflectance, bidirectional reflectance distribution function (*BRDF*) of diffuse samples, and both diffuse and non-diffuse transmittance. Samples up to 30 cm by 30 cm can be measured. The instrument and its characterization are described.

## 1. Introduction

The Optical Technology Division of the Physics Laboratory at the National Institute of Standards and Technology has designed and constructed a new reflectometer-spectrophotometer to replace the previous generation Reference Reflectance Instrument (RRI) [1]. The new instrument, the Spectral Tri-function Automated Reference Reflectometer (STARR), delivers higher accuracy, greater speed of measurement, and greater functionality than the previous instrument. The STARR is capable of measuring spectral reflectance (specular and diffuse) at incidence angles up to 80° from sample normal, and observation angles from within 5° of the incident beam to 80° from sample normal. Spectral transmittance can be measured for incident and observation angles up to 80° from sample normal. The receiver (detector system) has an aperture stop, the area of which has been accurately measured, which remains at a fixed distance from the illuminated spot on the sample for all observation angles. This allows the STARR to make high accuracy bidirectional reflectance distribution function (*BRDF*) [2] measurements. In all operating modes, a sample up to 30 cm by 30 cm can be raster-scanned to measure sample spatial uniformity.

The key improvements over the RRI include greater mechanical stability, higher precision positioning, higher source system throughput, improved detector performance, larger sample holder, sample raster-scanning capability, increased measurement speed, and improved air filtering for a cleaner laboratory environment.

## 2. Reflectometer Design

**Fig. 1:**
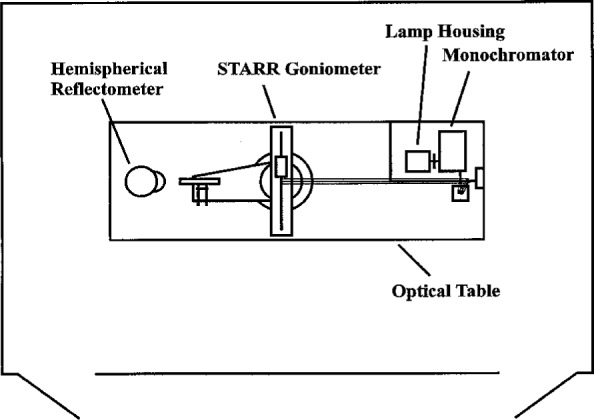
Top view of the Spectral Tri-function Automated Reference Reflectometer (STARR) facility.

Two major problems with the previous instrument (RRI) were poor mechanical stability and low signal levels in the ultraviolet (UV) spectral region. To ensure exceptional mechanical stability, heavy duty precision linear and rotary stages were used in the new design. All components of the STARR are mounted on a 3.6 m by 1.2 m by 0.3 m thick vibration isolated optical table. This table is located in the center of a light-tight room with black walls and ceiling. Special air filters have been incorporated into the room ventilation system to provide a clean environment. This room also houses all of the STARR electronic components. The instrument is fully automated and is controlled by a computer in an adjacent room. Figure 1 shows the source optics and monochromator mounted at one end of the optical table, the reflectometer-spectrophotometer near the center of the table, and a hemispherical reflectometer located at the far end of the optical table. This hemispherical reflectometer attachment, which is currently under development, will be used for measuring directional-hemispherical reflectance factor, and will not be discussed further in this paper.

**Fig. 2:**
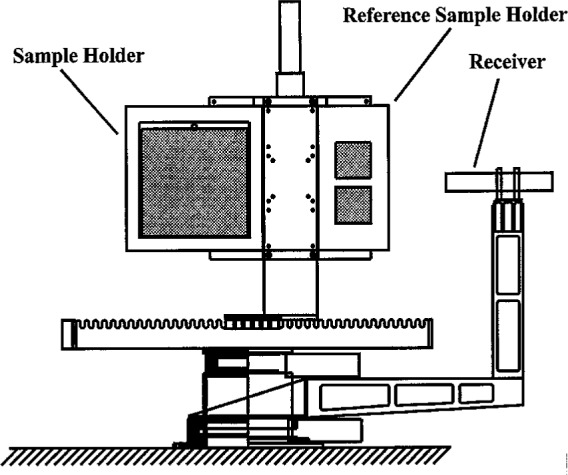
Front view of the STARR goniometer.

The STARR goniometer has four independent positioning stages. The sample holder, shown in Fig. 2, is attached to a two axis linear positioning system which gives the range of motion needed to raster-scan the entire 30 cm by 30 cm sample area, as well as to position either of two reference samples for measurement. This positioning system is mounted on a rotation stage used to rotate the sample with respect to the incident radiation. The relatively thin profile of the sample holder and positioning system gives this system its large working range of incident angles while maintaining rigidity. The receiver system is mounted on a light weight, rigid arm, which is mounted on a second rotation stage. This rotation stage is independent of and coaxial with the sample rotation stage. This allows the receiver to rotate around the sample holder at a constant radius of 672.6 mm (from the sample rotation axis to the receiver limiting aperture). These two rotation stages are precisely aligned. This is achieved through high precision machining of a heavy duty stainless steel chassis which was fabricated, along with most of the other custom components, in the NIST Fabrication Technology Division’s main shop. All four stages are fitted with servo motors controlled by a four axis motion controller.

To ensure adequate signal in the UV spectral region, the optical system was designed to minimize reflective losses. An optical system composed of *n* first surface reflective optics will have an overall effective reflectance (output flux divided by input flux, assuming no other loss mechanisms) of *ρ*_eff_ where

$$\rho_{\text{eff}}=\prod_{i=1}^{n}\rho_{i}$$

where *ρ*_1_ is the reflectance of the first element, and *ρ_n_* is the reflectance of the *n*th element. If we assume, for illustrative purposes, that all of the elements have the same reflectance *ρ*, then this simplifies to

$$\rho_{\text{eff}}=\rho^{n}.$$

Thus, if *n* is large, the overall effective reflectance of the optical system can be quite low, even for relatively high reflectance optics. The optical system for the RRI included 14 first surface reflective elements, while the STARR includes only 7 such elements. This provides a significant improvement in overall effective reflectance over the RRI.

## 3. Source System

The lamp housing (Oriel 7340 Monochromator Illuminator^1^) contains two sources. A 150 W ultraviolet enhanced xenon arc lamp is used for the 200 nm to 400 nm spectral range, and a 100 W quartz-tungsten-halogen (QTH) lamp is used for the 400 nm to 2500 nm spectral range. The xenon arc is monitored by a detector based light intensity controller (Oriel 68850 Light Intensity Controller) equipped with a UV bandpass filter to optimize the stability in this spectral region. This intensity controller provides feedback to the arc lamp power supply (Oriel model 68805) to stabilize the lamp intensity. The stability of the xenon arc was measured with and without the intensity controller. For this measurement, the receiver was positioned to measure the incident beam, and the beam and background were measured from 200 nm to 400 nm in 10 nm steps. This process was repeated ten times over a period of several hours. The intensity stabilizer improved the xenon arc stability by a factor of 5, reducing the relative experimental standard deviation of the measured intensity from 0.5 % to 0.1 %. The QTH lamp is operated by a high stability power supply (Oriel 68830 Radiometric Power Supply). The lamp housing has a collection mirror which focuses the radiation on the monochromator entrance slit, and matches the *f*/# of the monochromator. The collection mirror can be rotated to select either of the two lamps. The lamp housing is coupled to the 1/4 meter *f*/3.9 monochromator (Oriel 77700 Multispec 257) through a five position filter wheel. This filter wheel holds a series of cut-on (long-pass) filters, used to eliminate higher order wavelengths (order sorting). The monochromator has a four-grating turret equipped with three 600 lines per millimeter ruled gratings blazed at 200 nm, 400 nm, and 1000 nm, respectively, and a 150 lines per millimeter ruled grating blazed at 4000 nm (included for possible extension of the working range beyond 2500 nm). The filter wheel and the grating turret are controlled by the monochromator microprocessor. The filter and grating selection information, after being optimized experimentally, is stored in the monochromator nonvolatile memory. The proper filter and grating are automatically selected for any given wavelength. As the radiation exits the monochromator through a 1 mm circular aperture, it is collimated by a 51 mm diameter off-axis parabolic mirror. The radiation is then directed by a 51 mm diameter flat mirror through a series of baffles, an optical chopper, a Glan-Taylor polarizer, and a final baffle before exiting the source system along the optical axis of the instrument.

A 1 mm monochromator entrance slit is used for UV and visible (Vis) measurements. The spectral bandpass was evaluated using emission line measurements and found to be approximately 7 nm at 550 nm. A 2 mm entrance slit is used for near-infrared (NIR) measurements to improve signal to noise ratio, resulting in a slightly larger spectral bandpass in this region. A 1 mm diameter circular exit aperture is used for all measurements.

## 4. Receiver System

The STARR receiver system consists of a precision circular aperture with an area of 796.84 mm^2^ [3], a fused silica lens, and several baffles mounted in a 51 mm diameter cylindrical housing. The precision aperture is the limiting aperture of the receiver and defines the collection solid angle for BRDF measurements. The lens has two functions in this system. First, it condenses the nearly collimated 14 mm diameter beam onto the detector such that the detector is underfilled. Second, the lens images the sample plane of the goniometer onto the detector, giving the STARR receiver a well-defined field of view.

Two detectors are utilized in the STARR receiver. A 10 mm by 10 mm silicon photodiode is used for the spectral range of 200 nm to 1100 nm. A 1 mm diameter thermoelectrically cooled photovoltaic indium arsenide photodiode coupled to an averaging sphere is used for the spectral range of 900 nm to 2500 nm. The receiver system was constructed in such a way that the baffles, limiting aperture, and fused silica focusing lens remain fixed on the detector arm, while the detectors are interchangeable. This design permits the interchanging of detectors without the need to realign the system. A low noise transimpedance amplifier (TIA) is used with both detectors. When using the silicon photodiode, the output of the TIA is measured with a seven and one-half digit digital voltmeter (DVM). When using the indium arsenide photodiode, an optical chopper is used in conjunction with a lock-in amplifier. The output of the lock-in amplifier is measured with the DVM.

The linearity of the detectors was measured using a combination of multiple apertures and neutral density filters. A double aperture mechanism consisting of two adjacent semicircular apertures, each with its own shutter, was placed in the sample holder and positioned such that both apertures were overfilled by the incident beam. The signal due to light passing through aperture “A” was measured and designated *V*_A_. The signal due to light passing through aperture “B” was measured and designated *V*_B_. Finally, the signal due to light passing through both apertures simultaneously was measured and designated *V*_AB_. A neutral density filter was then used to attenuate the beam, and the above measurement sequence was repeated. This process continued using successively higher density filters until the attenuated beam could no longer be measured. These measurements were repeated at several wavelengths. The data were analyzed to determine at what signal level the relationship *V*_A_ + *V*_B_ = *V*_AB_ was no longer satisfied to within the uncertainty of the measurement. For both detectors, it was found that the above relationship was satisfied until the noise floor of the detector system was reached. That is, no measurable nonlinearity was found at signal levels above the noise floor.

Since the silicon photodiode is used without an averaging sphere, its response uniformity was measured in the NIST Detector Comparator Facility to determine if the detector was suitable for use in the STARR receiver. Table 1 shows the nonuniformity of this detector at four wavelengths. While the nonuniformity is as high as 1 % in the NIR and 0.6 % in the UV, because the detector is underfilled, and because these nonuniformity figures represent extreme values which are limited to very small portions of the detector active area, the effect of these nonuniformities is insignificant. Due to the imaging characteristics of the receiver system, this nonuniformity would only contribute significantly to the measurement error when measuring a sample with a significantly nonuniform spatial distribution of reflected energy, such as measuring near-specular *BRDF* of a mirror. This is not the type of measurement that this system was designed to perform. The nonuniformity of this detector will not contribute significantly to the measurement uncertainty for the types of measurements to be performed.

## 5. Measurement Results

For the purpose of this work, four types of samples were selected to demonstrate the capabilities of the new instrument: (1) A first-surface-aluminum-coated mirror, representative of the Standard Reference Material (SRM) 2003 used in industry to calibrate the photometric scale of spectrophotometers; (2) a porcelain enamel on steel tile which is used for colorimetric measurements of chromatic British Ceramic (BCRA) tiles; (3) an NG-9 black glass, representative of SRM 2026, which is also used to calibrate the photometric scale of spectrophotometers; and (4) a GL-2 black glass which was used to expand the dynamic range of the photomultiplier tube detector system used in the previous generation instrument for low level measurements in the ultraviolet region. All of these samples were chosen for their long history of stable, repeatable measurements.

Additionally, a freshly pressed plaque of Polytetrafluoroethylene (PTFE) resin was measured using an angle of incidence of 45° and angle of observation of 0° with respect to the sample normal (customarily denoted as 45°/0° geometry). This type of sample is currently being developed as a working standard for 45°/0° *BRDF* [4].

Measurements results given herein represent two types of measurement geometries: specular reflectance factor (*SRF*) using 6°/6° geometry, and bidirectional reflectance distribution function (*BRDF*) using 45°/0° geometry.

The *SRF* is measured by setting the angle of incidence (the angle between the incident radiation and the sample normal) to 6° and measuring the specularly reflected flux at 6° on the other side of the sample normal. The incident flux (*Φ*_i_) and the reflected flux (*Φ*_r_) are measured, and the ratio of these measurements gives the specular reflectance factor.

$$SRF=\Phi_{r}/\Phi_{i}.$$

*BRDF* is defined as the differential ratio of the reflected radiance (*L*_r_) to the incident irradiance (*E*_i_) and can be expressed as

$$BRDF=dL_{r}/dE_{i}$$

For practical measurements conditions [5], this can be expressed as

$$BRDF=\Phi_{r}/\left(\Phi_{i}\omega_{r}\cos\theta_{r}\right),$$

where *Φ*_r_ is the reflected flux collected by the receiver, *Φ*_i_ is the flux incident on the sample, *θ*_r_ is the angle of observation, and *ω*_r_ is the solid angle subtended by the limiting aperture of the receiver where, to a good approximation

$$\omega_{r}=\pi r^{2}/R^{2}$$

where *r* is the radius of the receiver limiting aperture and *R* is the distance from the sample to the receiver limiting aperture.

Table 2 lists the components of relative standard uncertainty, the relative combined standard uncertainties, and the expanded uncertainties for these measurements [6]. The incident and reflected flux measurements each have a relative standard uncertainty of 0.10 %. These values arise from statistical variations in repeated flux measurements made over a period of approximately 5 h using the xenon arc lamp and light intensity controller, and are due to lamp drift, amplifier noise and drift, voltage measurement noise, and other contributions. The receiver limiting aperture area was determined using the NIST High Accuracy Aperture Comparator, and its uncertainty was assigned based on the uncertainty budget of the comparator. The distance from the sample plane to the receiver limiting aperture was measured using a precision inside-micrometer. This distance was measured at five different detector arm angles at 45° intervals. This measured distance varied by 0.05 mm. The inside micrometer had a standard uncertainty of 0.05 mm. These values are small compared to the overall distance, so for simplicity these were added together and treated as a standard uncertainty of 0.10 mm, or 0.02 % of the distance. The uncertainty in the solid angle of detection was calculated by combining in quadrature the uncertainties in the receiver limiting aperture area and the distance from the sample plane to the receiver limiting aperture (with appropriate weighting factors). This yielded a relative standard uncertainty in the solid angle of detection of 0.075 %. To check the angular accuracy of the goniometer system, the receiver was centered on the incident beam and the receiver angle counter was set to 0°. A first surface mirror was then mounted in the sample holder and positioned so as to retro-reflect the incident beam. The sample angle counter was then set to 0°. The sample holder was rotated in 10° steps and the receiver was positioned to the appropriate (theoretical) angle to intercept the specular reflection of the beam. The distance from the center of the receiver to the center of the reflected beam was measured for each angular setting. This procedure was repeated several times. The largest measured displacement of the beam from the receiver center was 0.5 mm. Because the center of the beam could be estimated to 0.5 mm, this was added to the measured displacement for a maximum displacement of 1.0 mm. At a distance from sample to receiver of 672.6 mm, this corresponds to a relative standard uncertainty in the receiver angle of 0.09°. The rotary stages used in the STARR goniometer have an angular resolution of 0.001° and an angular standard uncertainty and repeatability of 0.01°. These are small compared to the measured receiver angular relative standard uncertainty of 0.09 %, and in fact, such stage-induced errors are included in the measurement of the receiver angular relative standard uncertainty.

**Fig. 3:**
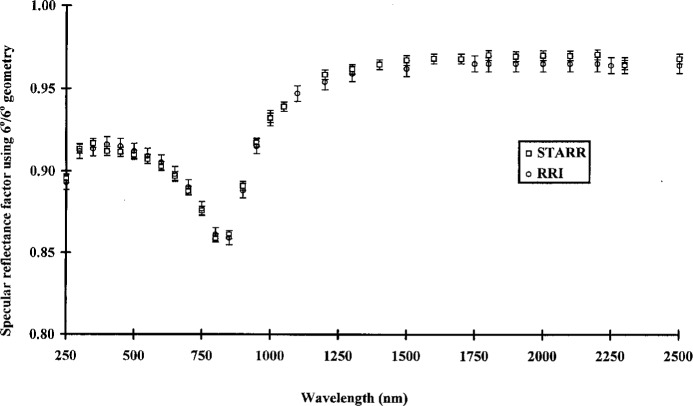
Specular reflectance factor of a first-surface-aluminum-coated mirror using a 6° angle of incidence and a 6° angle of observation (6°/6° geometry) as measured by the STARR and the Reference Reflectance Instrument (RRI).

Figure 3 shows the specular reflectance of an SRM 2003 first-surface-aluminum-coated mirror at 6°/6° geometry. Measurements made on the STARR and RRI agree to within their respective measurement uncertainties.

**Fig. 4:**
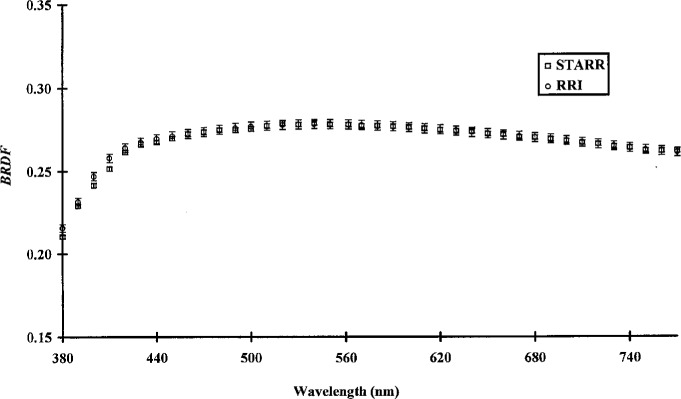
BRDF of a porcelain enamel on steel tile using a 45° angle of incidence and a 0° angle of observation (45°/0° geometry) as measured by STARR and by RRI.

Figure 4 shows the *BRDF* of a white porcelain enamel on steel plaque at 45°/0° geometry. It is interesting to note that the RRI data for this sample were obtained in 1977 while the STARR data were obtained in 1995. Measurements made on the two instruments agree to within their respective measurement uncertainties over most of the spectral range measured. Considering that nearly 20 years elapsed between these measurements, the agreement is remarkable, and demonstrates the excellent long term stability of this sample. The slight differences in the UV data could easily be due to aging of the sample or differences in cleaning techniques.

**Fig. 5:**
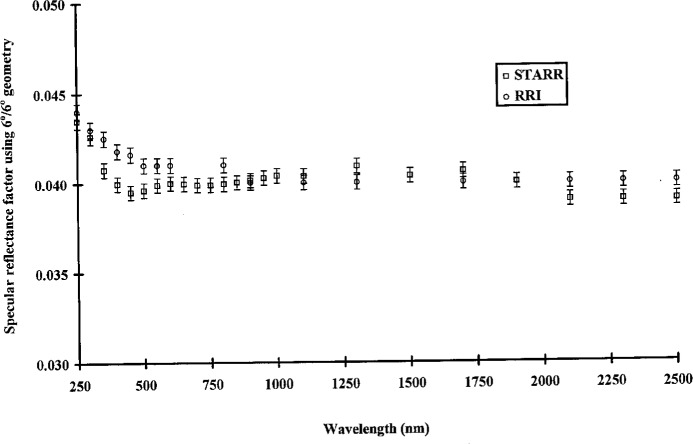
Specular reflectance factor of NG-9 black glass using 6°/6° geometry as measured by STARR and by RRI.

Figure 5 shows the *SRF* of NG-9 black glass (SRM 2026) at 6°/6° geometry. At some wavelengths the agreement between the instruments is excellent, while at other wavelengths they differ by several percent. Other types of black glass samples, such as GL-2, show much better agreement. A period of approximately 2 years elapsed between the RRI and STARR measurements, so further studies of NG-9 stability and the effects of cleaning procedures should be performed.

**Fig. 6:**
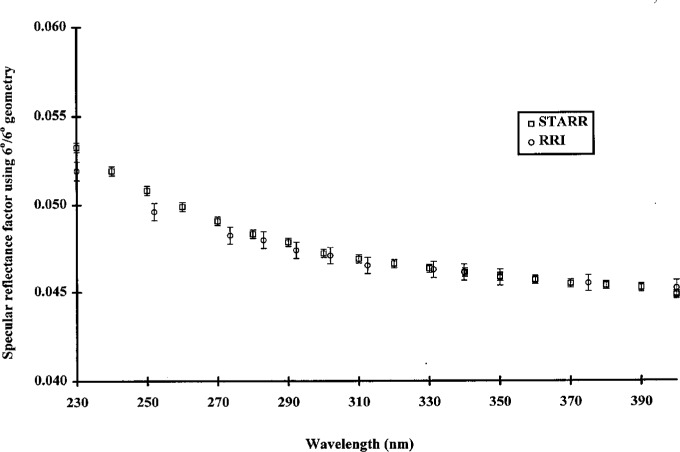
Specular reflectance factor of GL-2 black glass using 6°/6° geometry as measured by STARR and by RRI.

Figure 6 shows the *SRF* of GL-2 black glass at 6°/6° geometry. This sample was used in the RRI as a reference sample for low level reflectance measurements in the UV spectral region, and was chosen for its long measurement history and demonstrated long term stability. The agreement between the two instruments is excellent except for the 230 nm and 250 nm data. The RRI was known to have marginal low-level measurement performance at 250 nm. The RRI was not designed to operate at 230 nm, and at this wavelength, measurement performance was very poor.

**Figure 7:**
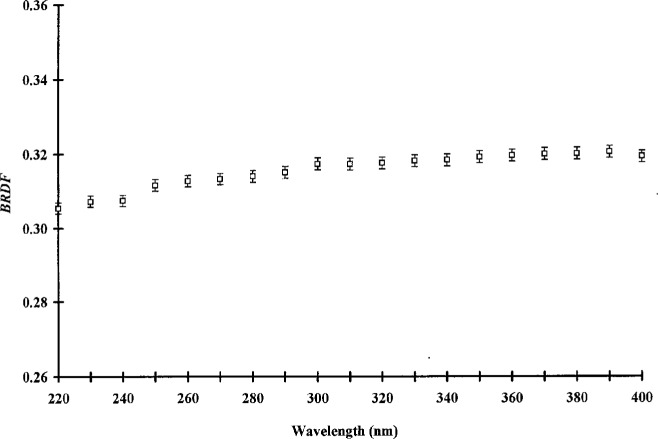
*BRDF* of pressed Polytetrafluoroethylene (PTFE) using 45°/0° geometry as measured by STARR.

Figure 7 shows the *BRDF* of pressed PTFE as measured by the STARR. Because this sample was freshly pressed for measurement on the STARR, no direct comparison can be made with the RRI. However, the results of the STARR measurements are in agreement with RRI measurements of similar samples.

## 6. Conclusion

The Reference Reflectance Instrument (RRI) had been in service at NIST for nearly 2 decades, performing specular reflectance and BRDF measurement services, as well as calibration of numerous SRMs. The Spectral Tri-function Automated Reference Reflectometer (STARR) was designed to replace the aged RRI, and to enhance the range of measurements that could be performed to include larger samples, raster-scanning of samples for uniformity, and spectral transmittance. The new instrument has been designed to provide these capabilities, as well as to provide greater speed of measurement, and more stable and accurate positioning.

The STARR source system was designed to deliver higher flux to the sample and more reliable alignment than the previous system. Care was taken in the optical layout to minimize the number of optical elements in the source system. The STARR source system (including the lamp housing, monochromator, and beam steering optics) has 7 first surface reflective elements versus 14 for the RRI. This allows the STARR source system to maintain optical performance longer in the presence of degradation of optical elements. As an example, if the reflectance of the elements degrades to 0.8, the effective reflectance of the seven elements would be 0.8^7^ = 0.202, while the same degradation in the RRI would result in an effective reflectance of 0.8^14^ = 0.044.

During a 1 year testing period of the STARR, the mechanical stability of the system was shown to be excellent. During this period, frequent checks of the optical alignment demonstrated that the instrument maintained proper alignment for extended periods. The system required re-alignment only as a direct result of making changes to the optical components to improve performance. The longest period between optical system changes was 6 months, and during this time, no alignment adjustments were needed.

The STARR receiver system was designed to give better detector performance over a broader spectral range than that of the RRI. The STARR can measure reliably at 200 nm, while the RRI could not perform below 250 nm. Even at 250 nm, the measurement performance of the RRI was marginal.

Two limitations of the STARR were found during testing. First, the Glan-Taylor polarizer has fairly low transmittance in the 200 nm to 350 nm spectral region. A UV grade polarizer will be evaluated for use in this spectral region. Second, while the indium arsenide phododiode has excellent responsivity and linearity, it is only available in sizes which are too small for use in the STARR receiver without some diffuser or light gathering mechanism. Several approaches to improve NIR performance are being considered. A 2 mm diameter InAs detector can be fabricated, but due to the relatively low shunt impedance of these detectors, it is not clear that the larger area will result in better performance. A nonimaging concentrator may be used to collect more flux from the averaging sphere and concentrate it onto the detector. Additional optical elements may be used to refocus the receiver at reduced image size directly onto the InAs photodiode, eliminating the need for the averaging sphere. Other detector technologies may also be evaluated.

The design goals for the STARR were to build an instrument which would perform all of the measurements of the former RRI, to perform these measurements with higher accuracy and greater speed, and to handle much larger samples. The STARR performs all of the measurements of the RRI, with additional capabilities, such as raster scanning of large samples, and transmittance measurements. Improvements in the detector system have reduced the radiometric uncertainties of these measurements. The accuracy of positioning and mechanical stability of the new system provide reduced geometrical uncertainties. High accuracy measurement of the receiver limiting aperture reduce both radiometric and geometric uncertainties. Many measurements which took several days on the RRI can be performed in a matter of hours on the STARR.

## Figures and Tables

**Table 1:** Silicon photodiode response non-uniformity

Wavelength	Detector response non-uniformity
250 nm	0.6 %
500 nm	0.2 %
800 nm	0.4 %
1000 nm	1.0 %

**Table 2:** Relative combined standard uncertainties, expanded uncertainties, and their components

Components of the relative standard uncertainties.					
Source of uncertainty				Relative standard uncertainty	
Incident flux measurement, *Φ_i_*				0.10 %	
Reflected flux measurement, *Φ*_r_				0.10 %	
Receiver limiting aperture area, *A*				0.05 %	
Distance from sample to receiver, *R*				0.02 %^a^	
Solid angle of detection, *ω*_r_				0.075 %	
Angle of observation, *θ*_r_				0.09°	
cos*θ*_r_	*θ*_r_ = 0°			<<0.001 %	
	*θ*_r_ = 15°			0.042 %	
	*θ*_r_ = 30°			0.091 %	
	*θ*_r_ = 45°			0.157 %	
	*θ*_r_ = 60°			0.272 %	
	*θ*_r_ = 75°			0.586 %	

Relative combined standard uncertainties and expanded uncertainties for coverage factor *k* = 2					

			*u*_c_(*SRF*)		*U*(*SRF*)

SRF measurements			0.14 %		0.28 %

			*u*_c_(*BRDF*)		*U*(*BRDF*)

*BRDF* measurements		*θ*_r_ = 0°	0.160 %		0.320 %
		*θ*_r_ = 15°	0.165 %		0.330 %
		*θ*_r_ = 30°	0.184 %		0.368 %
		*θ*_r_ = 45°	0.224 %		0.448 %
		*θ*_r_ = 60°	0.316 %		0.632 %
		*θ*_r_ = 75°	0.607 %		1.214 %

a This is the only type B component of the combined standard uncertainty. All other components are type A.

## Footnotes

1 Certain commercial equipment, instruments, or materials are identified in this paper to foster understanding. Such identification does not imply recommendation or endorsement by the National Institute of Standards and Technology, nor does it imply that the materials or equipment identified are necessarily the best available for the purpose.
